# The effects of the image attributes of golf star athletes supported by adult amateur golfers on sports participation desire and continuation intention

**DOI:** 10.3389/fpsyg.2025.1500592

**Published:** 2025-02-10

**Authors:** Ji-Hye Yang, Ga Young Kim, Hye Jin Yang, Chulhwan Choi, Chul-Ho Bum

**Affiliations:** ^1^Department of Human Movement Studies and Special Education, Old Dominion University, Norfolk, VA, United States; ^2^Department of Physical Education, Kyung Hee University, Yongin-si, Gyeonggi-do, Republic of Korea; ^3^Department of Physical Education, Gachon University, Seongnam-si, Gyeonggi-do, Republic of Korea; ^4^Department of Golf Industry, Kyung Hee University, Yongin-si, Gyeonggi-do, Republic of Korea

**Keywords:** sport star, amateur golfer, image attribute, sports participation desire, sports continuation intention

## Abstract

The image attributes of sports star athletes demonstrate positive effects on sports fans' psychological factors. In the context of golf, fans are also influenced by the players they support, experiencing a range of emotions. Therefore, this study aimed to examine the effects of the image attributes of golf star athletes—supported by adult amateur golfers—on the desire for sports participation and the intention to continue. This study involved 356 adult amateur golfers in Korea (253 male, 103 female). Questionnaires were used to measure participants' perceptions of image attributes, their desire for sports participation, and their intention to continue participating in the sport. The data were analyzed using descriptive statistics, validity and reliability, and multiple regression analyses. First, the image attributes factors (physical appearance, performance ability, and moral image) all had a significant effect on both the desire for sports participation and the intention to continue participating in the sport. However, performance ability did not impact affective desire. Second, while cognitive and behavioral desires significantly influenced the intention to continue participating, affective desire did not have an effect. In conclusion, fans identify diverse athletes' images (e.g., physical appearance, performance, and personality) through attending or watching sports games, and positive sports star image attributes enhance sports fandom, participation desire, and continuation intention. The results of this study indicate a close relationship between golf fans' perceptions of players' image attributes and their psychological factors. These findings could help improve fans' quality of life and provide valuable insights for understanding their fandom.

## 1 Introduction

Sports stars have been considered athletes with high sports skills and appeal in individual sports fields (Kim and Oh, 2017). They are usually exposed to the public through various media (Nam, 2019), and they sometimes become ideal people among the public (Kim and Choi, 2019) because they are respected physically, economically, and socially. Moreover, they sometimes become national heroes in mega sporting events such as Olimpic or the World Cup (Podoler, 2023). Thus, issues related to sports stars are enough to attract attention, and this attention is a significant driving force in the sports industry.

Watching sports stars is a common leisure activity (Ryu and Heo, 2016). This activity can be categorized into two types: attending and watching (Apostolou and Zacharia, 2015; Wenner, 2002). Both types of participation offer positive leisure benefits to individuals (Lyu and Lee, 2013). For example, participants can obtain sports team information (Oshimi and Harada, 2013) and experience reduced stress or improved social abilities (Eastman and Land, 1997). Despite the impact of COVID-19 in recent years, viewer ratings, which reflect the public's high value placed on sports, have remained high (Grix et al., 2021).

Watching sports can also have significant effects, such as helping individuals recognize athletes' images through opportunities to observe them (Ferrand and Pages, 1999). For example, participants can perceive athletes' seriousness and honesty during a game (Park et al., 2019), their performance skills (Kim et al., 2017), and personal traits such as friendliness, all of which are important factors in shaping athletes' image attributes. Kim et al. (2015) reported that an athlete's image significantly impacts not only the public's perception but also the athlete's own satisfaction.

Golf emerged later than other sports did, but professional golfer Se-ri Park, an icon for overcoming the International Monetary Fund (IMF) crisis in Korea (Kim et al., 2006), and the significant performances of other golfers have contributed to the growth of participants and fans (Choi and Bum, 2020). Notably, golf fans typically walk and watch games, which distinguishes them from other sports. This behavior reflects outstanding physical activity (Murray et al., 2017) and provides valuable experiences and memorization (Hwang and Lee, 2018), leading to a high number of visitors to golf courses each year.

Athletes can become sports stars when they capture people's hearts, even if they do not possess the highest level of current ability. Additionally, numerous fan clubs have recently been established based on Korea Ladies Professional Golf Association (KLPGA) athletes (Lee, 2019), and fan sentiment significantly enhances people's interest and enjoyment (Choi and Won, 2014). Furthermore, Watanabe et al. (2013) investigated the reasons why people wish to spend more time on golf courses. Their findings demonstrated that both affection for the player and for the game contribute to this desire, indicating a close relationship between players and their fans.

Currently, the government recognizes the importance of physical health and encourages people to start exercising (Mills et al., 2019). However, Choi (2009) emphasized that it is crucial for individuals to voluntarily engage in exercise when they find it interesting and enjoyable. Various factors contribute to the enjoyment of sports, but among them, fans can positively influence the image of both the athletes and the sport itself through watching sports (Li et al., 2018). Therefore, given that Tiger Woods' mental resilience, performance ability, and ethical integrity are widely acknowledged and highly regarded by many people (Dixon, 2002), it is anticipated that individuals form their perceptions of athletes on the basis of these attributes. Moreover, Kim H. K. et al. (2020) and Kim J. H. et al. (2020) demonstrated that attributes of athletes' image, such as appeal and professionalism, significantly influence people's behavior. Consequently, the image of sports stars is expected to have a significant effect on sports participation.

According to Jouper and Hassmén (2009) and Rodrigues et al. (2020), creating motivation is important for continued sports participation. Different types of motivation include social motivation, which involves participating in sports with others (Song and Park, 2015), and aesthetic motivation, which is related to anticipated physical changes, among other factors. In contrast to these motivations, Shin and Kim (2021) argued that people need to improve their personal autonomy and intrinsic motivation for continued sports participation. In addition, in the case of sports fans, their affection for athletes and sports teams that they support is considerable (Dwyer et al., 2015). For example, Delia et al.'s (2021) study verified that athletes play a significant role in enhancing their fans' quality of life; thus, athletes can expect sports fans to engage in sports voluntarily and continuously.

The image of sports stars has already attracted attention in the field of business administration, particularly in relation to media, advertising effects, and consumption (e.g., Ferreira et al., 2022; Malik et al., 2018). Recently, however, scholars have also begun to focus on research related to sports psychology (e.g., Zhang and Kim, 2020). Therefore, this study investigated the effects of the image attributes of golf star athletes supported by adult amateur golfers on sports participation desire and continuation intention, and the findings could contribute to the development of data that positively affect amateur golfers' overall quality of life.

### 1.1 Sports star image attribute

The fundamental meaning of an image, which encompasses an overall impression derived from a comprehensive perspective, involves individuals recognizing entities such as people or objects (Dichter, 1985). In the sports field, numerous previous studies have examined various images, such as the images of different sport types (e.g., Devlin et al., 2016) and sports corporate images (e.g., Zhou et al., 2020). The image of a sport star has such a significant impact that it can determine the perceptions of the other types of images mentioned above. For example, the impact is amplified when sports stars possess not only high-performance abilities but also appealing attributes such as appearance, personality, and a sense of humor (Kim J. H. et al., 2020). Moreover, sports stars are often viewed as ideal people by highly dedicated fans (Lines, 2001). Consequently, it is not unusual for the image of a sports star to influence and change the perceptions of other images. Additionally, recent examples indicate that the image of sports stars is increasingly seen not only as an economic tool but also as having a positive impact on fans' psychological and emotional wellbeing. For example, Kim and Choi (2019) reported that a sports star image significantly enhances fans' positive self-esteem, whereas Ahn (2018) reported that this image positively influences the public's confidence in sports.

### 1.2 Desire for sport participation

Sports participation has been recognized to have positive physical and psychological effects on participants through numerous previous studies (e.g., Diaz et al., 2019; Jenkin et al., 2018; Neely and Holt, 2014). Furthermore, Park and Kim (2015) defined sports desire as the motivation that individuals want to attend in sports, which is closely related to intrinsic motivation (Ryan, 1995). In the sports field, sports fans frequently experience a sense of identification when watching sports games (Asada et al., 2020), which can also increase their desire to participate in sports. Additionally, Grima et al. (2017) reported that sports participation desire is influenced by various factors, both internal and external. Lee (2008) further divided sports participation desire into three components. First, cognitive desire involves individuals acquiring information or skills through external sources. Second, affective desire leads to changes in individuals' emotional states, as they experience emotions similar to those of sports stars (Hyman and Sierra, 2010). Third, behavioral desire refers to actual participation in sports. Furthermore, Oh and Kim (2014) reported that the image of sports stars has a significant effect on the desire to participate in sports.

### 1.3 Sports continuation intention

According to Lee (2022), sports continuation intention is defined as individuals' determination to perform sports. This sport continuation intention is related to psychological needs and self-determination motivation (SDT). For example, Ryan and La Guardia (2000) reported that motivation decreases when basic psychological needs such as autonomy, competence, and relatedness are not met. On the basis of this theory, Deci and Ryan (2000) divided self-determination into amotivation, extrinsic motivation, and intrinsic motivation. For example, Kim and Lee (2009) reported that factors contributing to sustained sports participation include social interaction, enjoyment, and self-efficacy, encompassing both external and internal aspects. Moreover, sports fans often experience emotions such as identification with the teams or athletes they support. This emotional connection has a positive psychological effect (Wann, 2006) and could be considered one of the motivations for sports continuation intention. The hypotheses proposed in this study are as follows:

H1: The image attributes of golf star athletes supported by adult amateur golfers have a significant effect on the desire for sport participation.

H2: The image attributes of golf star athletes supported by adult amateur golfers have a significant effect on the continuation intentions of these athletes.

H3: Sports participation desire has a significant effect on sports continuation intention.

## 2 Methods

### 2.1 Study participants and data collection

The study was conducted with 391 adult amateur golfers in Korea to achieve the research objectives. Data were collected from 6 February to 26 August 2024 using an online survey. Participants were selected through a convenience sampling method, and voluntary oral agreements were obtained from all participants. After the responses were collected, 356 were ultimately analyzed, with 35 incomplete or unfaithful responses excluded. Table 1 presents the participants' demographic characteristics (gender, age, highest level of educational attainment, employment status, and frequency of playing golf).

**Table 1:** Results of social demographic information.

**Demographic**		**Frequency**	**Percentage (%)**
Gender	Male	253	71.1
	Female	103	28.9
Age	20s	81	22.8
	30s	130	36.5
	40s	108	30.3
	50s	30	8.4
	60 above	7	2.0
Highest level of educational achievement	High school below	27	7.6
	Bachelor below	266	74.7
	Graduate school above	63	17.7
Employment	Non-job	20	5.6
	Home maker	26	7.3
	Office job	106	29.8
	Professional job	100	28.1
	Service job	35	9.8
	Other	69	19.4
Frequency of playing golf	1–2 times per week	166	46.6
	3–4 times per week	138	38.8
	5–6 times per week	31	8.7
	Almost every day per week	21	5.9
Total		356	100

### 2.2 Measures

The study used questionnaires to examine the effects of sports star image attributes on sports participation desire and continuation intention. The image attribute scale, which was originally based on Ohanian (1990) and Choi (2002) and subsequently modified by Gown (2014), was evaluated via 12 questions. Additionally, the scale for sports participation desire, which was adapted from socialization theory in sports participation by Kenyon and McPherson (1973) and used in Lee (2008), was also modified for this study and comprises 12 questions. Finally, the sports continuation intention scale, which is based on the planned behavior theory of Ajzen and Driver (1992) and the sport commitment model of Scanlan et al. (1993) and used in Suh (2020), was adapted for this study and consists of five questions. The questionnaire utilized a Five-point Likert scale ranging from 1 = strongly disagree to 5 = strongly agree. A detailed description of all measures, including their structure, items, validity, and reliability, is provided in Section 3.

### 2.3 Statistical data analysis

IBM SPSS Statistics version 29.0 was used for the statistical analysis. First, descriptive statistics were used to analyze the participants' demographic information. Second, exploratory factor analysis was conducted to assess the validity of the collected data and to confirm the subfactors of the three variables (image attributes, sports participation desire, and sports continuation intention). Third, to verify the reliability of the collected data, reliability analysis was performed on the basis of Cronbach's alpha. Finally, multiple regression analysis was used to determine the effects of image attributes on sports participation desire and continuation intention. All analyses were performed with a significance level set at *p* < 0.05.

## 3 Results

Exploratory factor analysis was performed to verify the validity of the image attributes, sports participation desire, and sports continuation intention. Moreover, principal component analysis was conducted to extract factors, and the varimax method was used as a factor rotation method. As a result, both image attributes (physical appearance, performance ability, and moral image) and sports participation desire (cognitive desire, affective desire, and behavior desire) were divided into three subfactors with 12 items, and sports continuation intention was one factor with five items.

In the case of reliability analysis, Cronbach's alpha was used for verification, and the reliability of all the factors ranged from 0.919 to 0.592 (physical appearance: α = 0.837, performance ability: α = 0.839, moral image: α = 0.878, cognitive desire: α = 0.832, affective desire: α = 0.592, behavioral desire: α = 0.782, sport continuation intention: α = 0.919). According to Koo and Li (2016), a Cronbach's alpha coefficient exceeding 0.5 is considered sufficient to verify reliability. Therefore, the results are expected to be reliable. Detailed information on these results is provided in Tables 2–4.

**Table 2:** Result of factor analysis for image attributes.

**Items**	**1**	**2**	**3**
Moral image 11	**0.891**	0.197	0.154
Moral image 10	**0.856**	0.232	0.141
Moral image 12	**0.759**	0.176	0.254
Moral image 9	**0.736**	0.214	0.180
Physical appearance 3	0.195	**0.827**	0.137
Physical appearance 4	0.140	**0.785**	0.141
Physical appearance 2	0.256	**0.776**	0.231
Physical appearance 1	0.253	**0.742**	0.245
Performance ability 6	0.131	0.146	**0.878**
Performance ability 5	0.194	0.175	**0.857**
Performance ability 8	0.219	0.241	**0.754**
Performance ability 7	0.444	0.333	**0.517**
Eigenvalues	5.799	1.441	1.366
Variance (%)	48.326	12.012	11.387
Cronbach's alpha	0.878	0.837	0.839

Primary loadings for each observed variable are in bold.

**Table 3:** Result of factor analysis for sport participation desire.

**Items**	**1**	**2**	**3**
Cognitive desire 17	**0.796**	0.240	0.094
Cognitive desire 15	**0.776**	0.199	0.159
Cognitive desire 14	**0.773**	0.155	0.111
Cognitive desire 16	**0.714**	0.027	0.197
Cognitive desire 13	**0.565**	0.540	0.110
Behavioral desire 22	0.056	**0.850**	0.066
Behavioral desire 23	0.151	**0.809**	0.086
Behavioral desire 24	0.310	**0.678**	0.251
Affective desire 21	−0.033	0.004	**0.855**
Affective desire 20	0.323	0.185	**0.685**
Affective desire 19	0.316	0.213	**0.395**
Affective desire 18	0.348	0.324	**0.367**
Eigenvalues	4.752	1.329	1.126
Variance (%)	39.604	11.077	9.384
Cronbach's alpha	0.832	0.782	0.592

Primary loadings for each observed variable are in bold.

**Table 4:** Result of factor analysis for sport continuation intention.

**Items**	**1**
Sports continuation intention 28	**0.922**
Sports continuation intention 27	**0.910**
Sports continuation intention 29	**0.880**
Sports continuation intention 26	**0.836**
Sports continuation intention 25	**0.815**
Eigenvalues	3.815
Variance (%)	76.295
Cronbach's alpha	0.919

Primary loadings for each observed variable are in bold.

### 3.1 Multiple regression analysis

First, regarding the effects of image attributes on sports participation desire, physical appearance (β = 0.212, *t* = 4.062, *p* < 0.001), performance ability (β = 0.305, *t* = 5.716*, p* < 0.001), and moral image (β = 0.223, *t* = 4.239, *p* < 0.001) had significant positive effects on cognitive desire. Furthermore, physical appearance (β = 0.267, *t* = 4.613, *p* < 0.001) and moral image (β = 0.230, *t* = 3.925, *p* < 0.001) had significant positive effects on affective desire, whereas performance ability did not have a significant effect. In terms of behavioral desire, physical appearance (β = 0.300, *t* = 5.518, *p* < 0.001), performance ability (β = 0.175, *t* = 3.125, *p* < 0.001), and moral image (β = 0.212, *t* = 3.654, *p* = 0.002) had significant positive effects. Second, with respect to the effect of image attributes on sports continuation intention, physical appearance (β = 0.189, *t* = 3.147, *p* = 0.002), performance ability (β = 0.173, *t* = 2.812, *p* = 0.005), and moral image (β = 135, *t* = 2.221, *p* = 0.027) revealed positive effects. Finally, regarding the effect of sports participation desire on sports continuation intention, only cognitive desire (β = 0.161, *t* = 2.867, *p* = 0.004) and behavioral desire (β = 0.446, *t* = 8.361, *p* < 0.001) were significant. Detailed information on the results is presented in Tables 5–9.

**Table 5:** The effect of image attributes on cognitive desire.

**Dependent variable**		***B***	**SE**	**β**	***T***	***p***
Cognitive desire	Physical appearance	0.328	0.080	0.212	4.082	< 0.001^***^
	Performance ability	0.473	0.083	0.305	5.716	< 0.001^***^
	Moral image	0.302	0.071	0.223	4.239	< 0.001^***^
*F* = 71.148, *R^2^*= 0.377, Adjusted *R^2^*= 0.372						

^***^p < 0.001.

**Table 6:** The effect of image attributes on affective desire.

**Dependent variable**		***B***	**SE**	**β**	***t***	***p***
Affective desire	Physical appearance	0.347	0.075	0.267	4.613	< 0.001^***^
	Performance ability	0.087	0.078	0.067	1.127	0.260
	Moral image	0.262	0.067	0.230	3.925	< 0.001^***^
*F* = 34.364, *R^2^*= 0.227, Adjusted *R^2^*= 0.220						

^***^p < 0.001.

**Table 7:** The effect of image attributes on behavioral desire.

**Dependent variable**		***B***	**SE**	**β**	***t***	***p***
Behavioral desire	Physical appearance	0.205	0.037	0.300	5.518	< 0.001^***^
	Performance ability	0.120	0.038	0.175	3.125	0.002^**^
	Moral image	0.120	0.033	0.202	3.654	< 0.001^***^
*F* = 54.227, *R^2^*= 0.316, Adjusted *R^2^*= 0.310						

^**^p < 0.01; ^***^p < 0.001.

**Table 8:** The effect of image attributes on sports continuation intention.

**Dependent variable**		***B***	**SE**	**β**	***t***	***p***
Continuation intention	Physical appearance	0.268	0.085	0.189	3.147	0.002^**^
	Performance ability	0.247	0.088	0.173	2.812	0.005^**^
	Moral image	0.168	0.076	0.135	2.221	0.027^*^
*F* = 23.945, *R^2^*= 0.169, Adjusted *R^2^*= 0.162						

^*^p < 0.05; ^**^p < 0.01.

**Table 9:** The effect of sports participation desire on sports continuation intention.

**Dependent variable**		***B***	**SE**	**β**	***t***	***p***
Continuation intention	Cognitive desire	0.148	0.052	0.161	2.867	0.004^**^
	Affective desire	−0.044	0.059	−0.040	−0.744	0.458
	Behavioral desire	0.928	0.111	0.446	8.361	< 0.001^***^
*F* = 44.204, *R^2^*= 0.274, Adjusted *R^2^*= 0.267						

^**^p < 0.01; ^***^p < 0.001.

## 4 Discussion

This study examined the effects of the image attributes of golf star athletes supported by adult amateur golfers on sports participation desire and continuation intention. The results revealed that image attributes (physical appearance, performance ability, and moral image) had positive effects on cognitive desire, affective desire, and behavioral desire, but only performance ability did not influence affective desire. Furthermore, in the case of sports continuation intention, all subfactors of the image attribute had significant positive effects. Finally, both cognitive desire and behavioral desire, but not affective desire, significantly influence sports continuation intention.

Cognitive desire is influenced by image attributes (physical appearance, performance ability, and moral image). This is because people usually obtain and share information on the internet and social media. For example, Phua (2010) reported that sports fans can find detailed and statistical information online, and they can share this information on social media by using hashtags (Kim et al., 2021). Thus, sports fans usually can recognize their favorite teams and players' information or issues. Moreover, Kang (2015) highlighted that sports fans engage in communication with one another, allowing for the widespread sharing of overall perceptions about sports teams or athletes. Ultimately, the image attributes of sports teams or athletes can serve as catalysts for individuals' desire to both share information with others and seek out additional details.

Physical appearance and moral image had positive effects on affective desire. Affective desire is closely tied to individuals' psychological factors, such as emotions. Physical appearance significantly influences others' perceptions (Borah and Rankin, 2010), and an individual's overall physical appearance and impression can elicit positive emotions. Furthermore, sports fans often experience a strong sense of identification with athletes (Potter and Keene, 2012). According to Moital et al. (2019), affective desire includes many emotions, such as excitement and enjoyment, feeling special and proud, fear or missing out, regret, and embarrassment. However, it is not only related to athletes' performance. For example, Bum et al. (2022) reported that fans' self-efficacy and resilience improved even when the performance of the players or the team they supported was not good. Thus, fans' affective desires can undeniably be affected by more emotional situations, such as moral image, than performance.

Behavioral desire was positively influenced by physical appearance, performance ability, and moral image. According to Bang and O'Connor (2022), fans, as highly enthusiastic supporters, want to experience the time spent with the person they support. Moreover, Stavros et al. (2014) reported that many sports fans reduce their stress and boredom by watching or attending sports and can directly observe athletes' appearance, performance, and personalities. Thus, a positive sports star image can be expected to improve sports fans' behavioral motivation, such as wanting to play sports or watch games.

Additionally, physical appearance, performance ability, and moral image significantly affect sports continuation intention. This is related to sports fandom, which can be influenced by several factors. Gantz and Lewis (2023) noted that sports fandom is a common form of daily leisure around the world. Consequently, many people engage in sports fandom and form perceptions of individual athletes, but these perceptions can increase or decrease on the basis of the image of sports teams or players. For example, Kwon and Koo (2022) demonstrated that negative behavior by athletes can damage a team's image and reduce fandomness. Conversely, a positive image of sports teams or athletes enhances fans' continuation intention.

Finally, sports participation desire (cognitive desire, affective desire, and behavioral desire) positively influences sports continuation intention. Essentially, desires drive motivation, and sustained motivation leads to continued behavior. For example, An et al. (2024) reported that individuals who frequently participate in sports tend to continue sports more than others do in sports activities. Furthermore, Kim (2011) demonstrated that fundamental needs such as desire significantly positively affect both sports participation motivation and adherence intention in the sports field. From an international perspective, He (2023) used Korean samples and found that positive image attributes of sports athletes enhance fans' sports values and participation. Similarly, in Mahmoudian et al. (2021) and Magano et al. (2024)'s studies, the authors examined USA and Portugal sports fans' loyalty according to athlete image. As a result, there were close relationships between the two variables. Thus, similar results can be expected from sports fans worldwide, regardless of national differences.

On the basis of these results, athletes' image attributes positively influence both sports participation desire and sports continuation intention and sports participation desire also significantly affects sports continuation intention. Additionally, these findings indicate that athletes' image, sports participation desire, and continuation intention are closely related and that these data can be used to increase the quality of life of adult amateur golfers.

## 5 Conclusions and limitations

The study investigated the effects of the image of golf stars on the desire for sport participation and the intention to continue sports. The results of this study revealed that individuals' positive perceptions of sports, their participation, and their intention to continue participating are closely related. As evidenced by this, adult amateur golfers have positive images of athletes because they support them and wish for their success in the sports field. Furthermore, fans often recognize athletes' image on the basis of players' physical appearance, performance, and personality through watching or attending games. Despite occasional feelings of disappointment or frustration, sports fans with strong and dedicated fandoms generally do not easily alter their image or trust in athletes.

In addition, in the past, many sports fans preferred to watch games online despite COVID-19 (Thibaut et al., 2023), and Hoekman et al. (2024) reported that the percentage of sports participation increased recently compared with that during the COVID-19 period. This means that sports fans usually desire to watch and attend games despite being in an uncomfortable situation. Furthermore, Choi (2023) reported that people are more likely to continue participating in sports when they experience positive emotions in sports environments. Consequently, the study showed the same results: sports fans' positive and continuous emotions contribute to their enjoyment of the game and help them improve their quality of life.

There are some limitations in this study. First, this study focused on adult amateur golfers. However, there are various types of sports (e.g., soccer, basketball, and baseball), so further study should be conducted with more comprehensive sport types of fans. Second, this study investigated only the effect of positive athletes' image. Therefore, it would be important to conduct a follow-up study on the effects of negative athletes' image on sports fans' psychological factors. Third, the study focused only on aspects of sports fans' perceptions of athletes. Therefore, it is necessary to examine athletes' self-perceptions and the effect of these self-images on their psychological factors. Finally, this study relied on a cross-sectional design, which limits the ability to infer causal relationships. A longitudinal study could provide more insights into how athletes' image attributes influence sports participation desire and continuation intention over time.

## Data Availability

The original contributions presented in the study are included in the article/supplementary material, further inquiries can be directed to the corresponding authors.
